# The Relationship between the Transmission of Different SARS-CoV-2 Strains and Air Quality: A Case Study in China

**DOI:** 10.3390/ijerph20031943

**Published:** 2023-01-20

**Authors:** Ruiqing Ma, Yeyue Zhang, Yini Zhang, Xi Li, Zheng Ji

**Affiliations:** 1 School of Geography and Tourism, Shaanxi Normal University, Xi’an 710119, China; 2 International Joint Research Centre of Shaanxi Province for Pollutants Exposure and Eco-Environmental Health, Xi’an 710119, China

**Keywords:** air pollution, COVID-19, virus variants, generalized additive model, meteorological factors

## Abstract

Coronavirus Disease 2019 (COVID-19) has been a global public health concern for almost three years, and the transmission characteristics vary among different virus variants. Previous studies have investigated the relationship between air pollutants and COVID-19 infection caused by the original strain of severe acute respiratory syndrome coronavirus 2 (SARS-CoV-2). However, it is unclear whether individuals might be more susceptible to COVID-19 due to exposure to air pollutants, with the SARS-CoV-2 mutating faster and faster. This study aimed to explore the relationship between air pollutants and COVID-19 infection caused by three major SARS-CoV-2 strains (the original strain, Delta variant, and Omicron variant) in China. A generalized additive model was applied to investigate the associations of COVID-19 infection with six air pollutants (PM_2.5_, PM_10_, SO_2_, CO, NO_2_, and O_3_). A positive correlation might be indicated between air pollutants (PM_2.5_, PM_10_, and NO_2_) and confirmed cases of COVID-19 caused by different SARS-CoV-2 strains. It also suggested that the mutant variants appear to be more closely associated with air pollutants than the original strain. This study could provide valuable insight into control strategies that limit the concentration of air pollutants at lower levels and would better control the spread of COVID-19 even as the virus continues to mutate.

## 1. Introduction

Since December 2019, the Coronavirus Disease 2019 (COVID-19) epidemic has become a global health concern. It has been a pandemic because of high transmission capability of the virus, as well as high mobility and mortality for almost three years [1]. Several severe acute respiratory syndrome coronavirus 2 (SARS-CoV-2) variants have appeared in the past, such as Alpha (B.1.1.7), Beta (B.1.351), Gamma (p.1), Delta (B.1.617.2), Omicron (B.1.1.529), and other variants, which made the pandemic harder to restrain worldwide. Delta (B.1.617.2) and Omicron (B.1.1.529) were listed as currently circulating variants of concern (VOCs) by the World Health Organization (WHO) (https://www.who.int/activities/tracking-SARS-CoV-2-variants, accessed on 15 June 2022). With the virus mutating faster and faster, the activities and stabilities of SARS-CoV-2 in the environment and their transmission and infection characteristics changed markedly. It is known that the transmissibility of the Omicron variant increased sharply [2], with a longer duration of infectiousness and higher rates of breakthrough infection and reinfection, resulting in it rapidly becoming the current globally dominant variant [3]. Although the Omicron variant in many countries has replaced the Delta variant, some researchers have proposed the possibility of recirculating the Delta variant [4] and even co-circulation in the future. Previous studies found that the Delta variant has stronger infectivity, a shorter incubation period, and a higher viral load [5,6,7]. It was reported that Delta-infected individuals had an increased risk of hospitalization or emergency care attendance compared to individuals with the Omicron variant [8]. Meanwhile, the vaccine’s effectiveness might be decreased due to the immune evasion caused by the Delta and Omicron variants [9]. Despite vaccination and drug treatment, the pandemic has not been well controlled, and with the mutation of the virus, the possibility of a COVID-19 outbreak still exists.

Several studies showed that there was a significant association between COVID-19 infection with the air pollution indicators all around the world, such as in China [10], the United States [11], the United Kingdom [12], Italy [13], and Austria [14], which was similar to other typical respiratory diseases [15,16]. It could indicate a potential risk of air pollutants on respiratory infectious diseases, including COVID-19. Although various studies have suggested a potential link between COVID-19 infection and exposure to air pollutants, almost all these studies were based on the original strain of SARS-CoV-2. However, few studies have been conducted focusing on the link between air pollutants and infection caused by different variants of SARS-CoV-2, which might be an indispensable part of the COVID-19 epidemiological study.

In our study, we focused on the outbreaks of COVID-19 in three cities in China. In these three outbreaks, the dominant virus strains were different and uncontrolled transmission occurred along with covert community transmission. Then, the strict city-wide lockdown policy was adopted and it was exempted only when the epidemic situation was under control. Thus, we aimed to explore the relationship between air pollutants and SARS-CoV-2 infection according to the different dominant virus variants in order to give some new sight into COVID-19 prevention and control.

## 2. Materials and Methods

### 2.1. Study Area and Data Collection

In this study, three typical outbreaks dominated by different SARS-CoV-2 strains were chosen, including the first outbreak generated by the original SARS-CoV-2 strain in Wuhan in December 2019, the Delta (B.1.617.2) variant in Xi’an at the end of 2021 and Omicron (B.1.1.529) variant in Shanghai in the spring in 2022 that dominated the second and third outbreaks, respectively.

Over the past three years, many cities in China experienced the invasion of COVID-19. However, these three periods have some common characteristics. First, a concentrated outbreak occurred followed by a quick spread through the population. Then, an extremely strict city-wide lockdown for a relatively long period was implemented. In addition, Wuhan, Xi’an, and Shanghai are first-tier cities in China, and such extremely strict city-wide lockdowns are rare. To control the spread of COVID-19 and protect public health, the above-mentioned restriction policies include stay-at-home orders, traffic control, online teaching, homeworking, and closing stores and restaurants, which greatly affected people’s regular lives. The Chinese government will not easily allow a similar city-wide lockdown for such a long time to ensure normal socio-economic order.

**Figure 1:**
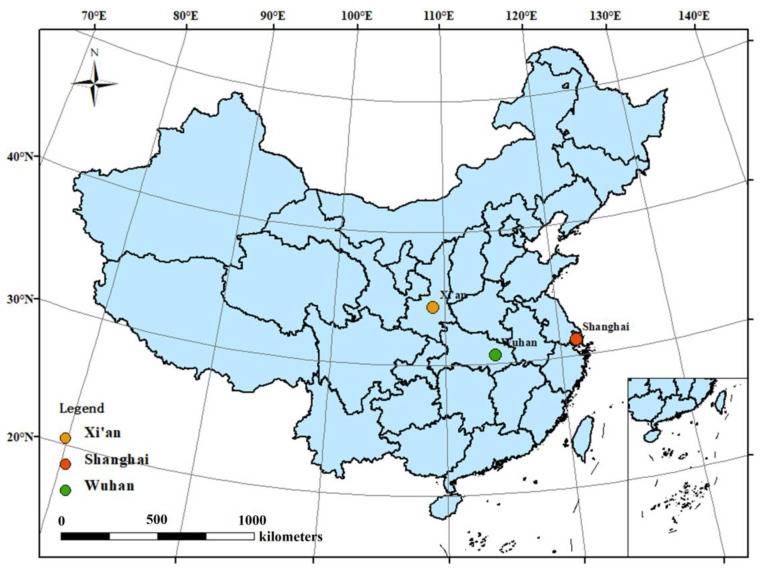
The geographical distribution of the study area in China includes Wuhan, Xi’an, and Shanghai.

Wuhan is located in central China (Figure 1) and is a transportation hub; it is also where the first COVID-19 confirmed case was reported publicly. On 12 February 2020, the number of new cases in a single day reached 13,436. In order to prevent the further spread of COVID-19, the government imposed a rare lockdown in Wuhan, which lasted from 23 January to 8 April in 2020.

Xi’an is the largest city in northwest China (Figure 1) and an important transportation junction. At the end of 2021, an outbreak of COVID-19 caused by the Delta (B.1.617.2) variant occurred in Xi’an. The consequences of this epidemic were severe, and a strict lockdown policy was adopted in Xi’an for more than a month. By 15 January 2022, there were 2044 confirmed cases in Xi’an. It should be noted that Xi’an was also the first city under lockdown because of the spread of the Delta (B.1.617.2) variant in China, especially after the nation-wide lockdown during the spring of 2021.

Shanghai is the economic and financial center of China. Similarly, in March 2022, an epidemic led by the Omicron variant attacked Shanghai. In a short period, the number of new asymptomatic infections increased dramatically, even up to 27,719 new cases on 13 April 2022. To prevent the further spread of the epidemic, the government of Shanghai also implemented a lockdown policy on 28 March 2022, and the lockdown state lasted for more than two months to contain the transmission of SARS-CoV-2. By 1 June 2022, there were a total of 649,379 confirmed cases caused by the Omicron variant in Shanghai.

**Figure 2:**
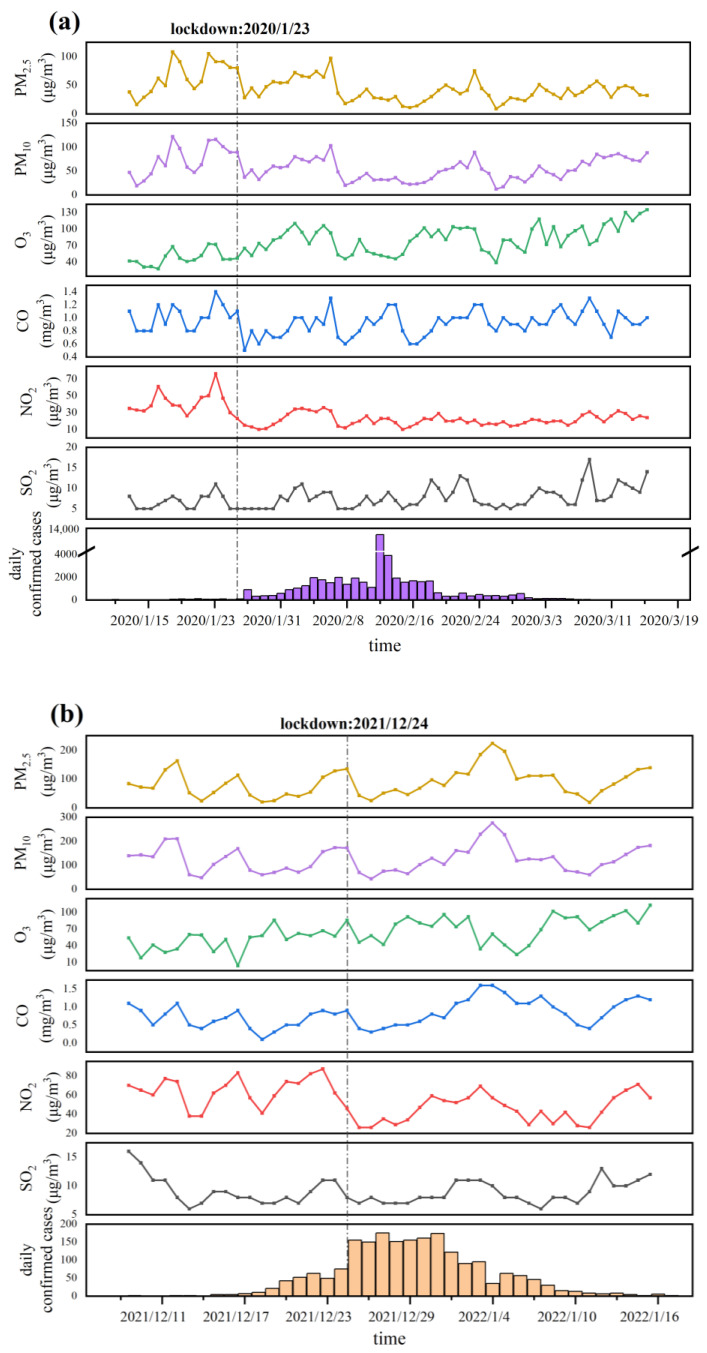

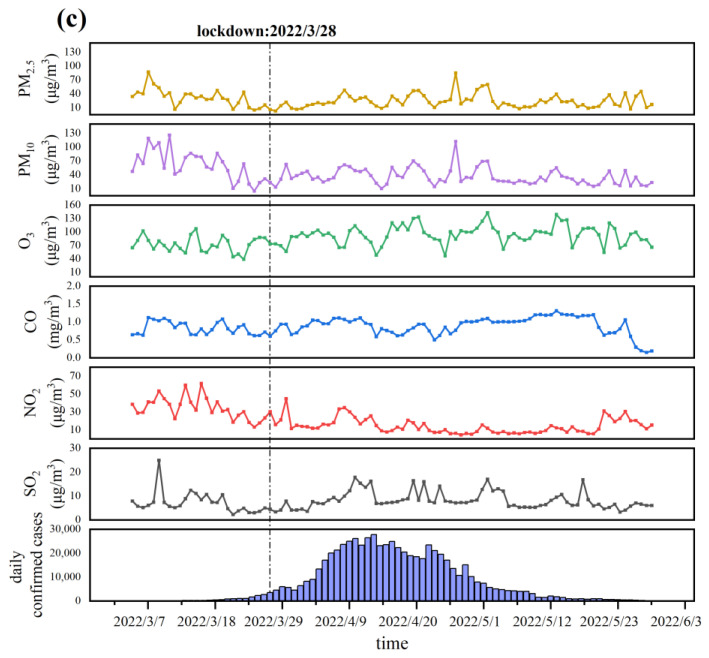
The number of daily confirmed cases in the three outbreaks and changes in the concentration of six air pollutants (PM_2.5_, PM_10_, NO_2_, SO_2_, CO, and O_3_). The vertical dot–dash lines in the figure refer to the time when the lockdown policy was implemented in the city. Note: (**a**) the first outbreak in Wuhan; (**b**) the second outbreak in Xi’an; (**c**) the third outbreak in Shanghai.

The concentrations of air pollutants (PM_2.5_, PM_10_, NO_2_, SO_2_, CO, and O_3_) and daily COVID-19 confirmed cases in the three outbreaks of the epidemic are shown in Figure 2. The data on the first outbreak were collected from 16 January to 17 March in 2020 in Wuhan, the data on the second outbreak were collected from 9 December 2021 to 15 January 2022, in Xi’an; the data on the third outbreak were collected from 1 March to 1 June in 2022 in Shanghai. The three phases involved in the study all implemented a lockdown policy when the spread of the epidemic reached an accelerated stage, on 23 January 2020, 24 December 2021, and 28 March 2022, respectively.

Daily new confirmed cases were reported by the National Health Commission of the People’s Republic of China (http://www.nhc.gov.cn/, accessed on 15 June 2022). Air pollutants include particles with diameters ≤ 2.5 μm (PM_2.5_), particles with diameters ≤ 10 μm (PM_10_), sulfur dioxide (SO_2_), nitrogen dioxide (NO_2_), ozone (O_3_), and carbon monoxide (CO). Daily data on these six air pollutants were obtained from the national city air quality real-time release platform (https://air.cnemc.cn:18007/, accessed on 15 June 2022)). Daily meteorological data were obtained from the National Meteorological Information Center (http://data.cma.cn, accessed on 15 June 2022)), including daily average temperature, relative humidity, precipitation, and wind speed.

### 2.2. Statistical Analysis

As the temporal and spatial distribution characteristics of air pollutants might be influenced by climate, season, and geographic region, the relationship between COVID-19 infection and exposure to air pollutants might be fuzzy and unclear; it is difficult to use a general parametric model to fit. Therefore, the flexible generalized additive model (GAM) could be a proper method [17] and applied in the research. Recently, various studies have used GAM to investigate the relationship between air pollution, meteorological factors, and COVID-19 infection [10,18,19,20]. GAM is a non-parametric regression model that uses a connection function to establish the relationship between response variables (COVID-19 confirmed cases) and non-parametric variables (air pollutants and meteorological factors), which could be better applied in the study to illustrate how air pollutants affect the transmission of COVID-19 [21].

The moving average method could be used to show the development direction and trend of events and then analyze the long-term trend of the prediction sequence, which was a helpful tool for analyzing time series. There is an incubation period in the process of virus infection. The incubation period is the time from infection occurring to the onset of symptoms [22], which is a crucial epidemiological parameter. The distribution of the incubation period was used in estimating the epidemic transmission potential [23]. Different SARS-CoV-2 strains showed a wide range of incubation times, ranging from 2.87 days [24] to 17.6 days [25]. It was shown that the mean incubation period was 6.4 days (95% CI: 5.6–7.7) for confirmed cases caused by the original strain [23] and 5.8 days (95% CI: 5.2–6.4) for the Delta variant [26], which was estimated by fitting the Weibull distribution with the Bayesian approach. The incubation period had a median of 3 days for both variants (Delta variant and Omicron variant) and the interquartile range was shorter for Omicron [27]. It was indicated that the weighted pooled mean incubation period of COVID-19 was 6.5 days (95% CI: 5.9–7.1) in a meta-analysis [22]. Therefore, the moving average method was adopted for air pollutants, which could solve the contradiction that the predicted value lags behind the actual observed value and better explains the relationship between air pollution exposure and COVID-19 infection. Concerning the potential lag effect of air pollution in affecting COVID-19 infection, several combinations of moving average concentrations of air pollutants (lag0, lag3, lag7) were calculated in this study. Here, lag0 indicates the same day, lag3 would then be the average over the same and previous 2 days (lag 0 to 2 days) and lag7 would then represent the average of the same and previous 6 days (lag 0 to 6 days). Furthermore, smoothing spline functions were used to flexibly fit independent variables into smooth curves to capture the influence of non-linear factors on dependent variables as much as possible. Modifying the model and finding a better degree of freedom in smoothing spline functions for the meteorological parameters in common is to benefit from the Akaike information criterion (AIC).

Spearman correlation analysis was performed to assess the correlation between air pollutants and meteorological factors so that the most significant correlated factors could be found via the correlation coefficients. *p* < 0.05 was considered statistically significant. To reduce the co-linearity, since some air pollutants were highly correlated, single pollutant models were used individually for the six air pollutants mentioned above [28].

In detail, the GAM with the Poisson regression family was applied to estimate the association between exposure to air pollutants and daily COVID-19 confirmed cases. The single air pollutant model was formulated as follows:

$$log\left(y_{t}\right)=Z_{i}+s\left(tem\right)+s\left(rh\right)+s\left(prcp\right)+s\left(win\right)+\mathrm{DOW}+day_{t}$$

In this single air pollutant model, *log*(*y_t_*) represents the log-transformed numbers of daily COVID-19 confirmed cases reported on day *t*. *Z_i_* is the moving average concentrations of different air pollutants, including 0-day moving average (lag0), 3-day moving average (lag3), and 7-day moving average (lag7), respectively. The meteorological factors include average temperature (tem), relative humidity (rh), precipitation (prcp), and wind speed (win). S (.) means the smoothing spline to control for the non-linear relationship, and meteorological parameters were fitted and expressed in the model as *s*(*tem*), *s*(*rh*), *s*(*prcp*), and *s*(*win*). The day of the week (DOW) was included in the models and coded as a dummy variable. In addition, *day_t_* is considered the effect of different control measures (lockdown or un-lockdown).

In this study, R Statistical software, Version 3.6.1 (R Foundation for Statistical Computing, Vienna, Austria) with the “mgcv” package (version 1.8–28) was used to perform data analysis. Spearman correlation analysis was performed by SPSS Statistics for Windows, Version 23.0 (IBM SPSS Inc., Armonk, NY, USA).

## 3. Results

### 3.1. Descriptive Analysis

As shown in Figure 2a, during the COVID-19 lockdown in mainland China in early 2020, the concentrations of five air pollutants (SO_2_, PM_2.5_, PM_10_, NO_2_, and CO) showed a decreasing trend, while O_3_ increased [29]. However, the sharp reduction in air pollutant concentrations might result from the quarantine order, traffic, and non-guaranteed industry restriction [30,31,32], which could only be maintained for a short period and would still return to normal levels in the long term [33].

The descriptive analysis of daily new confirmed cases of COVID-19, concentrations of air pollutants, and meteorological factors in three cities are shown in Table 1. Detailed data can be found in Tables S1–S3.

In the first outbreak in Wuhan, the daily average temperature, relative humidity, precipitation, and wind speed on average were 9.1 °C, 73.1%, 2.4 mm, and 2.4 m/s, respectively. In the second outbreak in Xi’an, the daily average temperature, relative humidity, precipitation, and wind speed on average were 3.7 °C, 52.5%, 0.1 mm, and 1.8 m/s, respectively. In the third outbreak in Shanghai, the daily average temperature, relative humidity, precipitation, and wind speed on average were 17.3 °C, 60.9%, 9.4 mm, and 2.5 m/s, respectively. It could be seen that the average value of the meteorological factors in the first outbreak (Wuhan) and the third outbreak (Shanghai) were much higher than those in the second outbreak (Xi’an). This might be due to different time–geographical characteristics. Shanghai and Wuhan are located at about the same latitude with a subtropical monsoon climate pattern, unlike the continental temperate monsoon climate of Xi’an. The first (Wuhan) and second (Xi’an) outbreaks of the epidemic occurred in winter and early spring, and the third outbreak (Shanghai) happened in the spring.

The average concentrations of PM_2.5_, PM_10_, SO_2_, and NO_2_ were 46.1 μg/m^3^, 58.1 μg/m^3^, 7.7 μg/m^3^, and 25.3 μg/m^3^ in the first outbreak (Wuhan), respectively; in the second outbreak (Xi’an), they were 87.3 μg/m^3^, 124.0 μg/m^3^, 8.9 μg/m^3^, and 53.3 μg/m^3^, respectively, and in the third outbreak (Shanghai), were 25.9 μg/m^3^, 42.9 μg/m^3^, 7.9 μg/m^3^, and 19.5 μg/m^3^, respectively. Noticeably, the O_3_ concentration in the third outbreak (Shanghai) was 87.7 µg/m^3^, higher than the 77.5 µg/m^3^ in the first outbreak (Wuhan) and the 63.4 µg/m^3^ in the second outbreak (Xi’an). The CO concentrations on average for both the first (Wuhan) and third (Shanghai) outbreaks were 0.9 mg/m^3^, higher than that in the second outbreak (Xi’an), which was 0.8 mg/m^3^. By comparing the pollutant concentrations during the three outbreaks, all concentrations of air pollutants in the second (Xi’an) outbreak, except for O_3_ and CO, were higher than those in the first (Wuhan) and third (Shanghai) outbreaks. As Xi’an is located in the Guanzhong basin in the northwestern part of China, there was a heating season in winter lasting for 4 months. During the heating season, fuel combustion and the meteorological and terrain conditions all played essential roles in the contribution of PM and other gaseous pollutant concentrations [34]. The meteorological conditions in winter were relatively stable. Moreover, the terrain of Xi’an was not conducive to the dispersal of air pollutants.

**Figure 3:**
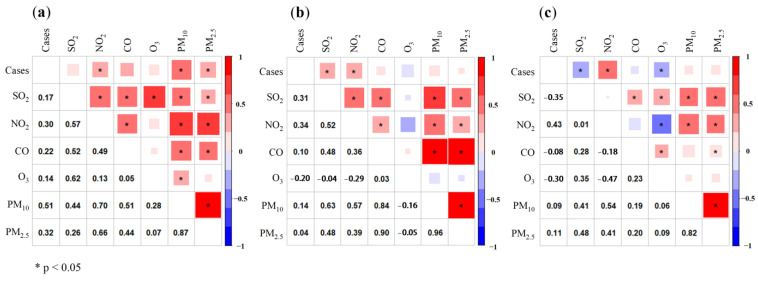
The correlation coefficients of Spearman correlation analysis between daily confirmed cases and six air pollutants in three outbreaks of an epidemic caused by different SARS-CoV-2 strains. Note: (**a**) Spearman correlation analysis in the first outbreak in Wuhan; (**b**) Spearman correlation analysis in the second outbreak in Xi’an; (**c**) Spearman correlation analysis in the third outbreak in Shanghai.

Air pollution significantly increases risk of disease, hospitalization, morbidity, and mortality worldwide [35]. Figure 3 shows the correlation coefficients of Spearman correlation analysis between daily confirmed cases and air pollutants in three outbreaks caused by different SARS-CoV-2 strains. The values of the Spearman correlation coefficients suggested how close the relationships might be between the concentration of air pollutants and COVID-19 infection, with the *p* value representing the level of significance for the coefficient (*p* < 0.05 was marked in the figure using *). In three outbreaks, confirmed cases were correlated with all air pollutants, but only a few pollutants and confirmed cases had significant linear correlations (as seen for existence of * in the first row of Figure 3). It indicated that the correlation between COVID-19 daily new confirmed cases and air pollutants were diverse. Consequently, a linear fit might not be sufficient to better fit the relationship between these factors. A GAM model that allows the fitting of complex nonlinear relationships might be more appropriate [36].

In the three outbreaks, most of these air pollutants showed significant correlations with each other. It can be found in Figure 3 that the strongest correlation exists between PM_2.5_ and PM_10_. It also indicated that SO_2_ was correlated with other pollutants, except O_3_ in the second outbreak and NO_2_ in the third outbreak. Co-linearity between many air pollutants was shown in this study. It also provides a basis for selecting variables in the GAM analysis. Therefore, to minimize the existence of co-linearity among the elements in the model, single pollutant models were conducted individually for the six air pollutants.

### 3.2. Relationship between Air Pollutants and COVID-19 Daily Confirmed Cases

**Figure 4:**
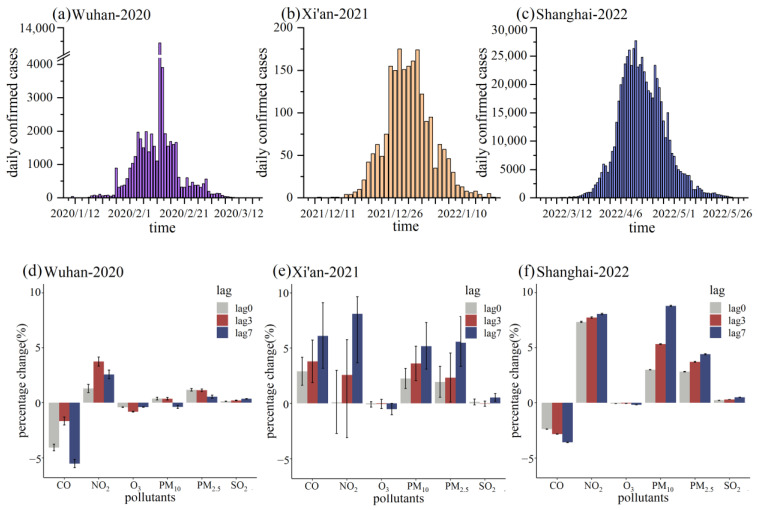
Percentage change (%) and 95% CI of daily COVID-19 confirmed cases associated with a unit increase in pollutant concentrations using single-pollutant models in three outbreaks of an epidemic caused by different SARS-CoV-2 variants (i.e., 1 μg/m^3^ increase in PM_2.5_, PM_10_, NO_2_, and O_3_, 0.1 μg/m^3^ increase in SO_2_ or 0.01 mg/m^3^ increase in CO). Note: confirmed cases in Wuhan were caused by the SARS-CoV-2 original virus in the first outbreak of the epidemic; confirmed cases in Xi’an were caused by the Delta variant in the second outbreak of the epidemic; confirmed cases in Shanghai were caused by the Omicron variant in the third outbreak of epidemic.

The result of GAM fitting is plotted in Figure 4. Among the selected three outbreaks, the longest epidemic season and the highest accumulated cases were found in Shanghai.

In the first outbreak (Wuhan), for the original virus strain transmission, positive associations between PM_2.5_, PM_10_, NO_2_, SO_2_, and COVID-19 confirmed cases, and negative associations between CO, O_3_, and COVID-19 confirmed cases were shown. It could be observed in Figure 4d that each one unit increase in PM_2.5_, PM_10_, NO_2_, and SO_2_ led to a 1.13% (95% CI: 1.01–1.24%), 0.36% (95% CI: 0.24–0.48%), 3.74% (95% CI: 3.33–4.15%) and 0.21% (95% CI: 0.18–0.23%) increase in daily confirmed cases, respectively. Meanwhile, a unit increase in CO and O_3_ was associated with a decrease of 1.67% (95% CI: 1.29–2.04%) and 0.83% (95% CI: 0.79–0.85%) in daily confirmed cases, respectively.

In the second outbreak (Xi’an), for the Delta variant transmission, positive associations between PM_2.5_, PM_10_, NO_2_, SO_2_, CO, and COVID-19 confirmed cases and negative associations between O_3_ and COVID-19 confirmed cases were indicated. It could be observed in Figure 4e that each one unit increase in PM_2.5_, PM_10_, NO_2_, SO_2_, and CO led to a 5.57% (95% CI: 3.34–7.85%), 5.18% (95% CI: 3.09–7.31%), 8.10% (95% CI: 3.79–9.98%), 0.52% (95% CI: 0.18–0.88%) and 6.09% (95% CI: 3.17–9.11%) increase in daily confirmed cases, respectively. Meanwhile, a 1 μg/m^3^ increase in O_3_ was associated with a 0.52% (95% CI: 0.01–1.03%) decrease in daily confirmed cases.

In the third outbreak (Shanghai), for the Omicron variant transmission, positive associations between PM_2.5_, PM_10_, NO_2_, SO_2_, and COVID-19 confirmed cases and negative associations between CO, O_3_, and COVID-19 confirmed cases which were found to be similar to that of the first one in Wuhan. It could be observed in Figure 4f that each one unit increase in PM_2.5_, PM_10_, NO_2_, and SO_2_ led to a 4.18% (95% CI: 4.12–4.23%), 8.80% (95% CI: 8.74–8.86%), 8.06% (95% CI: 8.00–8.13%) and 0.51% (95% CI: 0.50–0.51%) increase in daily confirmed cases, respectively. Meanwhile, a unit increase in CO and O_3_ was associated with a 3.59% (95% CI: 3.57–3.61%) and 0.18% (95% CI: 0.17–0.19%) decrease in daily confirmed cases, respectively.

By comparing the percentage change of daily confirmed cases due to exposure to per unit increase in the concentration of air pollutants, a general positive correlation could be found between most air pollutants and confirmed cases of COVID-19 caused by different SARS-CoV-2 strains, with the strongest correlation for PM_2.5_, PM_10_, and NO_2_. CO was positively associated with confirmed cases only in the second outbreak (Xi’an). In addition, different from other pollutants, there was a non-significant negative correlation between exposure to ozone and COVID-19 infection caused by different strains in three periods. It can be seen in Figure 4 that exposure to O_3_ has no clear effect size pattern with changing lags. It could be observed that the association between air pollutant concentrations in 7 days moving average (lag7) was more obvious than those in 3 days moving average (lag3) and lag0 in the second (Xi’an) and third (Shanghai) outbreak caused by Delta variant and Omicron variant, respectively. However, this trend seems to be not so applicable in the first outbreak (Wuhan) caused by the original strain.

**Figure 5:**
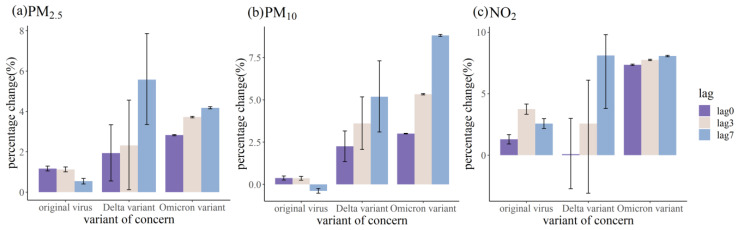
Percentage change (%) and 95% CI of daily COVID-19 confirmed cases due to exposure to increased unit concentrations of PM_2.5_, PM_10_, and NO_2_ in three outbreaks caused by different SARS-CoV-2 strains (i.e., 1 μg/m^3^ increase in PM_2.5_, PM_10_, and NO_2_).

To better illustrate the possible relationship between the COVID-19 infections caused by different strains and exposure to air pollutants, the percentage change in the number of confirmed cases due to exposure to the increased unit concentrations of PM_2.5_, PM_10_, and NO_2_ were compared together in Figure 5. It could be noted that the mutant variants appear to be more strongly associated with air pollutants (PM_2.5_, PM_10_, NO_2_) than the original strain. From Figure 5a,b, by comparing the percentage change of new cases (lag0 and lag3) for PM_2.5_ and PM_10_ with increased unit concentrations of exposure, it could be speculated that the sensitivity of the SARS-CoV-2 virus strain to particulate air pollutants is omicron > delta > original.

## 4. Discussion

Air pollution was the fifth major source of increased disease risk, hospitalization, morbidity, and mortality worldwide. The respiratory tract was a primary target of potential concurrent exposure to both air pollutants and airborne pathogens, including viruses [37]. Much research has been conducted concerning the relationship between air pollutants and COVID-19 infection caused by the original strain using the GAM fitting in different geographic regions [19,38,39]. However, our study focused on the link between concentrations of air pollutants and COVID-19 infection caused by different SARS-CoV-2 strains. The previous results indicated that short-term exposures to air pollution might be important aggravating factors for SARS-CoV-2 transmission and COVID-19 severity through multiple mechanisms [40]. Our results shown in Figure 5 suggested that people might be much more vulnerable to COVID-19 infection caused by the mutated variants of the SARS-CoV-2 virus (i.e., the Delta and Omicron in our study) with the exposure of air pollutants (i.e., PM_2.5_, PM_10_, NO_2_ in our study) than caused by the original strain. It might provide new insight into the current prevention and control of the COVID-19 pandemic.

It could be seen in Figure 4 that positive associations between exposure to most air pollutants (PM_2.5_, PM_10_, NO_2_, SO_2_) and COVID-19 confirmed cases were caused by different SARS-CoV-2 strains. It might indicate that exposure to air pollutants enhances individual susceptibility to COVID-19 [41]. According to the World Health Organization, environmental factors have a role in 35% of infectious disorders involving the lower respiratory tract, and SARS-CoV-2 infections were no exception [42]. Some studies found that there was a relationship between exposure to air pollutants (PM_2.5_, PM_10_, NO_2_, O_3_, SO_2_) and the increased prevalence of chronic obstructive pulmonary disease (COPD) [43] and the weakening of pulmonary function [44]. A study in the United States found that an increase of 1 μg/m^3^ in PM_2.5_ was associated with an 8% increase in the COVID-19 death rate (95% CI: 2–15%) [45]. Another study conducted in China showed that an increase of 10 mg/m^3^ PM_2.5_ and PM_10_ resulted in a 2.24% (95% CI: 1.02–3.46%), 1.76% (95% CI: 0.89–2.63%) increase in the daily counts of confirmed cases, respectively. Similar results could be found in Figure 4d. It could be found in Figure 4 that exposure to O_3_ has no clear effect size pattern with changing lags. The potential reason is that exposure to O_3_ has an acute and immediate influence and stimulates the symptoms of infected people, and the impacts gradually fade away during longer lagged periods [11]. The strong oxidizing and virucidal features of O_3_ may have been related to the non-significant negative correlation between short-term exposure to O_3_ and COVID-19 infection [46]. These data could demonstrate that environmental pollution increases individual susceptibility to COVID-19 on a global scale.

Among the six pollutants, exposure to PM_2.5_, PM_10_, and NO_2_ might significantly contribute to the susceptibility to COVID-19. Particle matter (PM), as a complex and variable mixture of particles and droplets suspended in the air, might initiate lung inflammation [47] and exposure to PM might enhance the susceptibility and severity of COVID-19 [48]. PM_2.5_ has been shown to be a vital transmission vector of pathogenic respiratory infections to the human alveolar epithelium [49]. Furthermore, PM_2.5_ provides a larger specific surface area for adsorption toxic chemical substances and pathogens [50]. Some studies confirmed the presence of the SARS-CoV-2 virus in atmospheric PM [51] and that individuals might be more susceptible to respiratory diseases due to inhalation and deposition of PM_2.5_ loaded with the virus [52]. Specifically, smaller particles such as PM_1_ were strongly associated with a higher incidence of COVID-19 than the effects of PM_2.5_ and PM_10_ under the experimental condition [18]. Therefore, the proportion of particles smaller than PM_2.5_ in the total particulate matter, as well as their composition, could not be neglected as well. It might explain our study’s positive percentage change of confirmed cases in exposure to PM_2.5_ and PM_10_. NO_2_ is a gaseous air pollutant generated mainly by vehicles, industrial production, and other combustion processes. Previous research indicated that NO_2_ as an airway irritant is potentially related to the immune system and might cause respiratory tract infections, promote lung inflammation [53], and sometimes even leads to mortality [54]. Recently, a study conducted based on the county scale in the United States showed a significant relationship between long-term exposure to NO_2_ and COVID-19 mortality [55]. In addition, exposure to NO_2_ affects the vulnerability of individuals to infectious diseases, including COVID-19. According to the available data, COVID-19 patients might have developed lymphopenia before the viral infection, which is strongly associated with short-term exposure to NO_2_ [56]. Moreover, the synergistic combination of ambient meteorological parameters (temperature and RH) and air quality (the toxicity of PM) might have a significant effect on the vitality and transmission of aerosol biological constituents [57]. Combining these chemicals and pathogens together, air pollution made it easier to cause human respiratory illnesses. Therefore, reducing the environmental burden of air pollutants might be seen as an important primary preventative intervention for reducing individual vulnerability to SARS-CoV-2 infection and COVID-19-related mortality.

It could be noted from Figure 5 that the mutant SARS-CoV-2 variants appear to be more closely associated with air pollutants (PM_2.5_, PM_10_, NO_2_) than the original strain. Different mutant variants existed in the environment [58]. Mutations found in VOCs were associated with increased transmissibility [2] and antibody escape [59]. Based on the evolution path of the virus, the dominant virus variants usually possess much stronger infectivity and are better adapted to the human immune system [60]. The R_0_ (average number of people that one sick person will infect) of the Delta variant was 5.08 [61], whereas the R_0_ of the Omicron variant raised to 11.88 [62], both much higher than the R_0_ of the original virus, which was 2.79 [63]. The transmissibility of the Delta variant was reported to have increased by 97% compared with the original virus [64], and the spread of the Omicron variant was even faster than the Delta variant [65]. Infection with the Delta variant markedly increased the risk of disease progression [6]. However, the clinical data suggested that the possibility of Omicron-infected individuals turning into severe cases was significantly reduced compared with individuals infected earlier with the Delta variant [66]. It might be caused by exposure to past variants, the effect of vaccine protection [60], and the behavioral habit of keeping a certain social distance. At the same time, there was a large number of asymptomatic cases, which might lead to the possibility of the virus spreading in a silent state without the patient being aware.

Recently, it was reported that there was an apparent trend toward increasing the positive electrostatic potential from the original virus strain through the Delta variant up to the Omicron variant [67]. Such change might be easier for the SARS-CoV-2 to bind to angiotensin-converting enzyme 2 (ACE2) [68], which has negative electrostatic surface potential patches [69]. Based on the interactions between positive and negative electrical charges, it indicated that the presence of aerosol particles could affect the atmospheric electrical conductivity in the near-ground layer [70], presumably leading to the above-mentioned easier binding. Furthermore, ACE2 would generate an anti-inflammatory peptide, and then the peptide might be over-expressed in the case of inflammation from PM exposure, thus increasing the probability of COVID-19 entering the cells [48]. Therefore, further research is needed to continue exploring the role of air pollution from this point of view. The neutralizing activity against SARS-CoV-2 variants was found to be significantly enhanced in those who had been fully vaccinated during the recovery phase after infection [71]. It implies that even if the virus was mutating, vaccination remains the most effective countermeasure to stop the disease from severity. Moreover, although it was reported that the pathogenicity of the Omicron variant has decreased, in developing countries, such as China, the number of medical beds per 1000 people was only 4.67 in 2020 [72]. Therefore, prevention and control of COVID-19 infection still need to be taken into consideration.

Many researchers have investigated the relationship between meteorological parameters, such as temperature and humidity, and virus survival under laboratory conditions [73] or in the real world [20,74]. Due to the temporal limitations of the three periods selected, such as the study period almost being in the same season, the temperature variation is very small therefore it may be difficult to observe a more pronounced trend within the limiting temperature period. The smoothing spline function was applied for the meteorological factors in the study, while lag structures analysis of average temperature and relative humidity were not included in the study which would make the results not robust enough. Since the contribution of meteorological parameters is not the focus of this study, the results are not presented in detail. The specific contribution of meteorological parameters to COVID-19 infection is not conclusive yet and should be focused on in future research.

Our study still has several limitations. First, COVID-19 as an infectious disease, and daily infection data tend to show a pronounced autocorrelation. The high autocorrelation of infectious diseases may lead to some biases in statistical inference, which makes the relationship between infectious diseases and other factors not robust enough. It is still necessary to find a more efficient approach and conduct more detailed studies on the lag structure to explore the influence of external factors on infectious diseases for the future. Second, an epidemic dominated by one virus strain involved only one outbreak, and perhaps the pool of data was not sufficient, and similar studies in other regions will be needed to further explore the relationship between mutant SARS-CoV-2 strains and air pollution. Simultaneously, for personal privacy reasons, there was no access to patient information to carry out subgroup analysis by gender and age to examine sensitive populations. Third, community and secondary transmission was a critical route of COVID-19 spread, and these often occur over very short distances, with an unclear relationship with weather conditions and air pollution. At last, our understanding of the potential role of air pollution in SARS-CoV-2 transmission was limited by the knowledge gap in some aspects, such as the resistance characteristics of the virus in the environment and the combined effect of multiple air pollutants with pathogens.

## 5. Conclusions

A statistical correlation between the percentage change in the number of COVID-19 confirmed cases and exposure to air pollutants was found. Among the six pollutants, exposure to PM_2.5_, PM_10_, and NO_2_ significantly contributed to the susceptibility to COVID-19. The mutant variants appear to be more strongly associated with air pollutants than the original strain. The study and prediction of such a relationship will help to combine better meteorological factors and air pollutants to observe the transmission characteristics of seasonal or geographic epidemics caused by viruses or other pathogens. As the virus mutates and the epidemic spreads, a comprehensive study of the transmission mechanisms of the virus is essential, and policies will be adjusted and updated accordingly.

Given the severity of the pandemic, there is a need to continue maintaining routine preventive measures such as social distancing, wearing masks, and washing hands, which could effectively slow down the spread of the pandemic. The direction of viral mutations was still unclear but limiting the concentration of air pollutants to lower levels and improving individual fitness would better control the spread of COVID-19 in the environment and promote public health.

## Figures and Tables

**Table 1:** Descriptive statistics of daily COVID-19 confirmed cases, air pollutant concentrations, and meteorological factors during three epidemic outbreaks (Wuhan: 16 January to 17 March in 2020; Xi’an: 9 December 2021, to 15 January 2022; Shanghai: 1 March to 1 June in 2022).

	Wuhan (68 Days)				Xi’an (44 Days)				Shanghai (99 Days)			
	Max	Min	Average	Median	Max	Min	Average	Median	Max	Min	Average	Median
Daily confirmed cases	3910	0	543.2	131	175	0	46.6	14	27,719	0	6559.4	1487
Relative humidity (%)	94.3	42.7	73.1	75.0	86.5	28.0	52.5	49.5	95.3	8.9	60.9	63
Precipitation (mm)	36.3	0	2.4	0	2.8	0	0.1	0	82.9	0	9.4	0
Wind speed (m/s)	5.6	1.1	2.4	2.2	3.3	0.8	1.8	1.85	4.4	0.7	2.5	2.5
Average temperature (°C)	21.5	0.7	9.1	8.6	8	−2.3	3.7	3.4	25.5	7.5	17.3	18.3
PM_2.5_ (μg/m^3^)	108	9	46.1	43	224	19	87.3	80	86.8	2.7	25.9	22.7
PM_10_ (μg/m^3^)	122	12	58.1	57	276	43	124.0	120.5	125.8	5.1	42.9	34.7
SO_2_ (μg/m^3^)	17	5	7.7	7	16	6	8.9	8	24.8	2.2	7.9	7.1
NO_2_ (μg/m^3^)	76	10	25.3	22	87	26	53.3	57	61.7	4.3	19.5	15.5
O_3_ (μg/m^3^)	135	28	77.5	78	113	4	63.4	60.5	142.5	38.9	87.7	88.8
CO (mg/m^3^)	1.4	0.5	0.9	0.9	1.6	0.1	0.8	0.8	1.3	0.1	0.9	0.9

## Data Availability

Data were provided in Supplementary Materials.

## Supplementary Materials

The following supporting information can be downloaded at: https://www.mdpi.com/article/10.3390/ijerph20031943/s1, Table S1: Data of daily COVID-19 confirmed cases, meteorological data (including relative humidity, precipitation, wind speed, and average temperature), and daily concentration of air pollutants (including SO_2_, NO_2_, CO, O_3_, PM_10_, and PM_2.5_) in Wuhan in the first wave of the epidemic; Table S2: Data of daily COVID-19 confirmed cases, meteorological data (including relative humidity, precipitation, wind speed, and average temperature), and daily concentration of air pollutants (including SO_2_, NO_2_, CO, O_3_, PM_10_, and PM_2.5_) in Xi’an in the second wave of the epidemic; Table S3: Data of daily COVID-19 confirmed cases, meteorological data (including relative humidity, precipitation, wind speed, and average temperature), and daily concentration of air pollutants (including SO_2_, NO_2_, CO, O_3_, PM_10_, and PM_2.5_) in Shanghai in the third wave of the epidemic.
