# Supplementary material for: The Relationship between the Transmission of Different SARS-CoV-2 Strains and Air Quality: A Case Study in China
Source: Int J Environ Res Public Health. 2023 Jan 20;20(3):1943. doi: 10.3390/ijerph20031943 (PMC9916065; doi:10.3390/ijerph20031943)
Supplement: Supplementary file 1 [file ijerph-20-01943-s001.zip › ijerph-2126223-supplementary.pdf]

Table S1. Data of daily COVID-19 confirmed cases, meteorological data (including relative humidity, precipitation, wind speed, and average temperature), and daily concentration of air pollutants (including SO_2, NO_2, CO, O_3, PM_{10}, and $PM_{2.5}$) in Wuhan in the first wave of the epidemic.

Date	Confirmed cases	Relative humidity (%)	Precipitation (mm)	Wind speed (m/s)	Average temperature (°C)	SO_2 (μg/m^3)	NO_2 (μg/m^3)	CO (mg/m^3)	O_3 (μg/m^3)	PM_{10} (μg/m^3)	$PM_{2.5}$ (μg/m^3)
2020/1/11	41	87.7349	0	1.314	2.527	5	38	0.8	32	44	39
2020/1/12	0	88.2286	0	2.763	2.863	6	61	1.2	28	80	62
2020/1/13	0	72.9433	0	2.459	5.723	7	47	0.9	51	61	49
2020/1/14	0	57.8638	0	3.25	5.554	8	39	1.2	68	122	108
2020/1/15	0	91.2496	9.7	2.663	1.05	7	38	1.1	47	97	91
2020/1/16	4	83.6217	0	2.4	1.617	5	26	0.8	41	58	60
2020/1/17	17	78.744	0	1.825	1.508	5	36	0.8	44	47	44
2020/1/18	59	81.2389	0	1.8	3.825	8	48	1	52	63	56
2020/1/19	77	73.9171	0	1.658	5.121	8	50	1	73	114	105
2020/1/20	60	62.0293	0	2.513	7.75	11	76	1.4	72	116	91
2020/1/21	105	87.5837	3.9	2.529	6.521	8	47	1.2	45	101	91
2020/1/22	62	91.0423	12.1	2.242	5.788	5	30	1	45	89	81
2020/1/23	70	87.9166	3.3	2.708	7.346	5	23	1.1	47	89	80
2020/1/24	77	91.3323	5.9	3.654	6.133	5	15	0.5	65	37	28
2020/1/25	46	88.4233	4.5	3.025	4.417	5	13	0.8	52	52	45
2020/1/26	80	83.7256	0.4	3.371	3.092	5	10	0.6	74	32	30
2020/1/27	892	80.1786	0	1.683	3.558	5	11	0.8	63	48	47
2020/1/28	315	74.8201	0	1.342	3.542	5	16	0.7	80	60	56
2020/1/29	356	76.2399	0	1.683	5.383	8	21	0.7	85	57	54
2020/1/30	378	61.9506	0	1.796	6.308	7	28	0.8	98	60	55
2020/1/31	576	53.1475	0	2.917	8.325	10	34	1	110	80	72
2020/2/1	894	57.5435	0	1.954	10.025	11	35	1	94	74	66

Date	Confirmed cases	Relative humidity (%)	Precipitation (mm)	Wind speed (m/s)	Average temperature (°C)	SO_2 (μg/m^3)	NO_2 (μg/m^3)	CO (mg/m^3)	O_3 (μg/m^3)	PM_{10} (μg/m^3)	$PM_{2.5}$ (μg/m^3)
2020/2/2	1033	76.4172	0	1.254	8.546	7	33	0.8	73	69	64
2020/2/3	1242	63.2496	0	1.663	7.817	8	31	1	94	80	74
2020/2/4	1967	66.6946	0	1.761	8.591	9	36	0.9	106	73	64
2020/2/5	1766	68.9757	9.3	4.029	8.725	9	32	1.3	93	103	97
2020/2/6	1501	89.5284	10	3.363	4.392	5	14	0.7	53	48	36
2020/2/7	1985	82.8963	0	1.746	4.821	5	12	0.6	46	20	18
2020/2/8	1379	80.498	0	1.058	5.246	5	17	0.7	53	26	23
2020/2/9	1920	74.3088	0	1.279	8.454	6	20	0.8	81	35	31
2020/2/10	1552	82.0336	0.4	1.708	9.467	8	26	1	60	45	43
2020/2/11	1104	86.5231	0	1.425	10.475	6	17	0.9	55	31	28
2020/2/12	13436	84.9205	0	1.9	10.904	7	23	1	52	32	27
2020/2/13	3910	85.3942	0	3.713	14.783	9	23	1.2	49	31	24
2020/2/14	1923	85.8546	20.4	5.6	11.071	7	18	1.2	46	36	30
2020/2/15	1548	93.0244	18.6	3.15	0.683	5	10	0.8	54	25	13
2020/2/16	1690	64.2519	0	1.371	3.142	6	13	0.6	78	22	11
2020/2/17	1600	55.3678	0	1.692	6.004	6	17	0.6	88	23	14
2020/2/18	1660	45.8095	0	2.1	10.021	8	23	0.7	102	26	22
2020/2/19	615	58.3155	0	2.142	9.538	12	22	0.8	86	34	30
2020/2/20	319	52.5777	0	2.746	12.333	10	29	1	98	48	41
2020/2/21	314	77.037	0.3	2.217	10.271	7	20	0.9	81	53	50
2020/2/22	599	62.803	0	2.588	13.25	9	20	1	104	57	43
2020/2/23	348	54.0876	0	2.8	16.467	13	23	1	101	69	35
2020/2/24	464	68.1915	0.1	2.238	19.492	12	18	1	103	57	41
2020/2/25	370	73.9827	36.3	3.746	15.229	7	21	1.2	100	89	75

Date	Confirmed cases	Relative humidity (%)	Precipitation (mm)	Wind speed (m/s)	Average temperature (°C)	SO_2 (μg/m^3)	NO_2 (μg/m^3)	CO (mg/m^3)	O_3 (μg/m^3)	PM_{10} (μg/m^3)	$PM_{2.5}$ (μg/m^3)
2020/2/26	383	78.6699	1.6	4.104	12.183	6	15	1.2	62	54	44
2020/2/27	313	89.8419	0.4	3.013	8.292	6	17	0.9	57	45	32
2020/2/28	420	94.3245	9.3	1.388	6.075	5	16	0.8	39	12	9
2020/2/29	565	85.9221	0	1.539	8.765	6	19	1	80	17	17
2020/3/1	193	72.8288	0	3.771	9.846	5	14	0.9	80	38	28
2020/3/2	111	79.7157	1	2.579	7.488	6	15	0.9	67	36	26
2020/3/3	114	76.5882	0.3	1.275	7.833	6	18	0.8	58	27	23
2020/3/4	131	62.4111	0	1.735	10.596	8	22	1	100	40	33
2020/3/5	126	58.6925	0	2.57	12.878	10	21	0.9	118	60	51
2020/3/6	74	78.732	0	1.333	12.592	9	18	0.9	72	48	41
2020/3/7	41	71.7346	0.3	2.404	15.242	9	20	1.1	104	42	34
2020/3/8	36	85.7072	13	3.263	14.863	8	20	1.2	68	32	27
2020/3/9	17	84.238	3.2	2.7	10.388	6	15	1	88	50	44
2020/3/10	13	68.3523	0	1.708	11.467	6	19	0.9	97	52	32
2020/3/11	8	62.1008	0	2.015	14.11	12	27	1.1	105	70	38
2020/3/12	5	76.4664	0.2	1.688	12.779	17	31	1.3	72	63	48
2020/3/13	4	75.0264	0	3.062	10.814	7	25	1.1	79	85	57
2020/3/14	4	63.8706	0	1.6	11.73	7	19	0.9	109	78	47
2020/3/15	4	49.7465	0	1.792	16.754	8	26	0.7	118	82	29
2020/3/16	1	57.4829	0	2.279	14.921	12	32	1.1	96	86	45
2020/3/17	1	58.5824	0	1.925	16.913	11	29	1	130	79	49

Table S2. Data of daily COVID-19 confirmed cases, meteorological data (including relative humidity, precipitation, wind speed, and average temperature), and daily concentration of air pollutants (including SO_2, NO_2, CO, O_3, PM_{10}, and $PM_{2.5}$) in Xi'an in the second wave of the epidemic.

Date	Confirmed cases	Relative humidity (%)	Precipitation (mm)	Wind speed (m/s)	Average temperature (°C)	SO_2 (μg/m^3)	NO_2 (μg/m^3)	CO (mg/m^3)	O_3 (μg/m^3)	PM_{10} (μg/m^3)	$PM_{2.5}$ (μg/m^3)
2021/12/6	0	43.4681	0	2.675	7.3	16	70	1.1	54	139	84
2021/12/7	0	35.1681	0	3.25	7.7	14	65	0.9	18	143	72
2021/12/8	0	56.4333	0	1.525	4.4	11	60	0.5	41	135	68
2021/12/9	1	65.9742	0	1.1875	4.7	11	77	0.8	28	209	132
2021/12/10	0	56.6957	2.8	0.8875	4.9	8	74	1.1	34	210	163
2021/12/11	0	50.4652	1	2.2	7.7	6	38	0.5	60	60	52
2021/12/12	1	42.3368	0	2.2125	5	7	38	0.4	59	48	24
2021/12/13	1	60.1506	0	1.9125	2.6	9	62	0.6	29	103	53
2021/12/14	0	62.3653	0	1.1375	4.6	9	70	0.7	51	136	85
2021/12/15	4	71.8426	0	1.075	3.8	8	83	0.9	4	169	113
2021/12/16	4	36.6621	0	1.9875	5.2	8	57	0.4	55	79	44
2021/12/17	7	31.4136	0	3.125	1.3	7	41	0.1	58	60	20
2021/12/18	10	31.2252	0	1.925	5.2	7	59	0.3	86	70	25
2021/12/19	21	36.3027	0	2.0125	6.3	8	74	0.5	51	88	48
2021/12/20	42	41.2802	0	1.875	6.9	7	72	0.5	62	71	40
2021/12/21	52	46.1244	0	1.225	5.2	9	82	0.8	58	94	55
2021/12/22	63	52.3835	0	1.4	4.2	11	87	0.9	67	156	106
2021/12/23	49	69.2743	0	1.475	3.4	11	62	0.8	57	173	128
2021/12/24	75	71.251	0	3.2	2.9	8	46	0.9	86	172	135
2021/12/25	155	63.3044	1.3	2.475	-1.7	7	26	0.4	46	70	43
2021/12/26	150	42.1569	0.3	1.8	-2.3	8	26	0.3	58	43	25
2021/12/27	175	58.3842	0	1.25	-2.2	7	35	0.4	42	75	51

2021/12/28	151	62.5224	0	2.325	8	7	29	0.5	79	80	63
2021/12/29	155	35.3225	0	1.225	1.2	7	34	0.5	92	64	46
2021/12/30	161	47.6059	0	0.8875	8	8	47	0.6	81	102	68
2021/12/31	174	51.9898	0	1.4625	1.9	8	59	0.8	75	129	97
2022/1/1	122	50.4552	0	2.25	3.35	8	54	0.7	96	104	78
2022/1/2	90	44.4177	0	1.9875	2	11	52	1.1	74	161	122
2022/1/3	95	55.5816	0	0.8125	2.05	11	57	1.2	92	154	117
2022/1/4	35	59.3653	0	1.525	1.1625	11	69	1.6	34	229	185
2022/1/5	63	70.1622	0	1.925	2.5375	10	57	1.6	61	276	224
2022/1/6	57	75.4876	0	2.8125	3	8	49	1.4	41	227	196
2022/1/7	46	76.9418	0	1.825	3.375	8	43	1.1	24	118	100
2022/1/8	30	86.4771	1	1.3	3.4375	7	29	1.1	40	126	111
2022/1/9	15	84.7867	0	1.95	2.825	6	43	1.3	69	123	111
2022/1/10	13	45.89	0	1.925	4.3	8	30	1	102	135	113
2022/1/11	8	48.4752	0	1.975	3	8	42	0.8	90	78	56
2022/1/12	6	28.0295	0	3.125	5.65	7	28	0.5	92	72	48
2022/1/13	8	33.7342	0	2.95	1.525	9	26	0.4	69	60	19
2022/1/14	4	40.8626	0	1.45	2.75	13	42	0.7	83	102	59
2022/1/15	1	45.2125	0	1.3625	4.7	10	57	1	94	114	82
2022/1/16	5	46.0194	0	1.325	4	10	65	1.2	103	145	107
2022/1/17	1	47.9852	0	1.675	3.4	11	71	1.3	81	174	133
2022/1/18	0	47.7742	0	1.3	3.2	12	57	1.2	113	182	139

Table S3. Data of daily COVID-19 confirmed cases, meteorological data (including relative humidity, precipitation, wind speed, and average temperature), and daily concentration of air pollutants (including SO_2, NO_2, CO, O_3, PM_{10}, and $PM_{2.5}$) in Shanghai in the third wave of the epidemic.

Date	Confirmed cases	Relative humidity (%)	Precipitation (mm)	Wind speed (m/s)	Average temperature (°C)	SO_2 (μg/m^3)	NO_2 (μg/m^3)	CO (mg/m^3)	O_3 (μg/m^3)	PM_{10} (μg/m^3)	$PM_{2.5}$ (μg/m^3)
2022/3/1	2	13.5522	76.2609	3.113	13.5522	6.0455	41.1364	1.1182	80.8182	119	86.8182
2022/3/2	8	10.425	57.4583	1.9125	10.425	7.4118	40.6471	1.0706	62	97.2353	61.4118
2022/3/3	16	12.6542	62.3333	2.2167	12.6542	24.8421	53.2632	1.0316	79.5789	109.3158	53.0526
2022/3/4	19	13.35	82.9167	1.7375	13.35	7.2941	44.8235	1.1	69.7059	54.1765	34.5294
2022/3/5	28	12.0458	57.375	2.8583	12.0458	5.6522	38.5217	1.0261	57.087	125.8261	42.0435
2022/3/6	48	8.9417	49.7083	2.575	8.9417	5.087	22.5217	0.8435	74.9565	41.6522	6.4783
2022/3/7	55	10.1625	58.75	1.7625	10.1625	5.9167	38.2917	0.9625	63.0417	48.75	21.1667
2022/3/8	65	12.1833	62.375	1.6708	12.1833	8.8261	59.7826	0.9609	53.1364	77.4286	39.3182
2022/3/9	80	13.8333	59.3333	2.9417	13.8333	12.3636	40.7727	0.65	94.5	86.6364	39.5
2022/3/10	75	15.7458	67.125	2.8417	15.7458	11.05	32	0.64	107.1	79.95	30.85
2022/3/11	83	18.5667	68.5417	1.4125	18.5667	8.3478	61.6957	0.8043	57.6087	78.6957	34.8696
2022/3/12	65	18.9625	77.125	2.7375	18.9625	10.619	45.1905	0.6476	54.1905	56.8095	27.5238
2022/3/13	169	21.6292	66.7917	2.4708	21.6292	7.381	29.2857	0.781	70.0952	51.9524	28.7619
2022/3/14	139	19.0583	70.0833	3.0042	19.0583	7.25	41.25	0.9833	66.5833	86.625	47.375
2022/3/15	202	60.4583	0	2.7083	15.5417	10.5455	30.8182	1.0773	92.2727	68.2727	30.2273
2022/3/16	158	69.2917	0	2.4917	19.6083	4.6957	32.6522	0.8087	80.4348	49.2174	26.8261
2022/3/17	260	92.2917	1.0333	4.3	15.8167	2.2083	18.5417	0.6833	44.3333	11	6.7647
2022/3/18	374	83.2917	0.0083	3.7167	9.2292	3.76	26.36	0.856	50.44	25.76	20.2222
2022/3/19	509	73.75	0	2.3	10.5292	4.9048	30.2381	0.919	38.9524	63.6667	43.1905
2022/3/20	758	84.9167	1.375	2.2458	7.5292	3.04	18.28	0.668	71.44	20.08	9.7826
2022/3/21	896	95.3333	1.9583	3.5875	8.0125	3	13.04	0.616	83.16	5.087	4.8571
2022/3/22	981	75.125	0.2375	2.2833	8.9917	3.52	17.64	0.62	87.56	23.2	8.0526

2022/3/23	983	57.5	0	2.3208	10.6125	5	23.3043	0.7174	86.9565	31.3043	15.6957
2022/3/24	1609	65.375	0	3.8458	12.2667	4.4167	30.25	0.6042	73.0417	23.5	5.8261
2022/3/25	2269	91.375	0.9958	4.4542	17.1042	3.3333	15.7917	0.75	73.25	13.6667	2.6667
2022/3/26	2676	63.875	0.0042	4.35	15.0375	4.1304	21.1304	0.9348	69	30.5217	14.3333
2022/3/27	3500	51.5417	0	1.6958	14.1917	7.7826	44.6087	0.9348	56.5652	62.6957	21.7826
2022/3/28	4477	58.7917	0	2.8125	11.7375	4.087	11.4783	0.6478	89.6087	32.2353	8.7857
2022/3/29	5982	60.9583	0	2.7167	13.2583	4.1304	15.087	0.6957	88.9565	38.3478	6.3043
2022/3/30	5653	69.125	0	2.6583	18.3417	4.5417	13.8333	0.8583	97.625	43.2917	8.125
2022/3/31	4502	72.8333	0.0667	3.2917	12.7583	3.56	13.4	0.892	89.96	47.56	14.75
2022/4/1	6311	50.6087	0	3.1348	11.113	7.6	11.8	1.0467	97.8667	30.4667	16.9333
2022/4/2	8226	55.9583	0	1.8375	10.9542	6.9231	12.0769	1.0385	103.7692	34.9231	20.6154
2022/4/3	9006	56.2917	0	2.0667	11.4333	6.75	16.25	0.95	92.75	24.5455	17.1667
2022/4/4	13354	60.25	0	2.2875	12.7333	8.2143	15.5	0.95	96.8571	29.5	21.3846
2022/4/5	17077	69	0	1.8375	13.275	9.3571	18.0714	1.1	88.0714	33.6429	20.3571
2022/4/6	19982	62.875	0	1.3458	16.7417	7.8182	33.0909	1.1091	65.2727	55	33.2727
2022/4/7	21222	57.75	0	1.5917	19.3417	9.9231	34.7692	1.0692	65.4615	61.6923	47.8462
2022/4/8	23624	46.75	0	3.0792	20.3583	12.2	29.9333	1	102.8	57.6667	34.4
2022/4/9	24943	49.2917	0	3.0417	20.4958	17.7857	24	1.0571	113.5	49.0714	24.7143
2022/4/10	26087	67.8333	0	3.1208	20.9417	15.3333	16.5833	1.1083	99.4167	46.9167	30.6667
2022/4/11	23342	59.7917	0	2.1292	24.2833	13.6154	21.3846	0.9615	87.1538	52	32.6923
2022/4/12	26330	63	0	2.2167	25.0167	16.1333	25.5333	0.9267	77	38.2667	22.1333
2022/4/13	27719	90.2083	3.375	3.6083	15.5125	6.8667	14.7333	0.5867	48.1333	21.7143	13.3077
2022/4/14	23072	93.75	0.8583	4.0875	14.1583	6.8	8.8667	0.8067	65.6667	10.4	8.6667
2022/4/15	23513	63.625	0	3.3667	14.8417	7.1333	7.6	0.76	88.4667	19.7692	13.7692
2022/4/16	24820	40.5833	0	2.3	14.9042	7.3077	9.1538	0.7077	120.0769	56.25	34.6923
2022/4/17	22248	46.5	0	1.9708	14.9958	7.6	13	0.6133	105.3333	38.3571	26.4

2022/4/18	20416	51.1667	0	1.8125	16.3458	8.3571	10.7143	0.6357	120.0714	34.5	16.2143
2022/4/19	18901	60.7083	0	1.6042	16.8958	8.8571	20.6429	0.7571	104.6429	55.3571	34.8571
2022/4/20	18495	56.25	0	2.5208	18.7958	16.4	17.8667	0.8333	130.1333	69.8667	46.6
2022/4/21	17629	70.25	0.1792	2.5917	19.6	8.2	10.4667	0.9333	133.0667	60.8667	47.0667
2022/4/22	23370	65.2917	0	1.9542	22.5	15.9286	17.1429	0.9357	98.5	48.4286	36
2022/4/23	21058	91.5417	1.775	1.4667	19.1125	7.7857	9.3571	0.75	90.8571	28.8	21.0714
2022/4/24	19455	81.875	0	2.1125	19.3042	7.1333	7.1333	0.5	84.0667	14.2727	10.3333
2022/4/25	16980	87.75	0.4333	2.6375	21.2958	14.1333	7.4	0.62	81.2667	29.6154	21.4
2022/4/26	13562	90.0417	0.1125	2.0208	20.2292	7.8667	10.2	0.8533	46.2	24.875	23.3333
2022/4/27	10622	58.9583	0.0083	3.0667	18.7792	7.5333	5.8	0.6667	100.3333	48.3571	27.5
2022/4/28	15032	78.0417	0.5125	2.3958	17.6625	7.0667	6.2	0.7667	83.8667	111.9333	84.6429
2022/4/29	10181	76.0833	0.3458	3.2417	14.2333	7.2667	4.3333	0.9733	102.5333	25.7857	18.3333
2022/4/30	7872	67.4583	0	1.9042	14.7167	7.2143	6.0714	1.0143	99.5	34.5385	28.5
2022/5/1	7333	57.5833	0	2.2333	16.275	7.8667	5.0667	1	99.3333	33.1429	26.0667
2022/5/2	5669	59.5833	0	1.4958	17.9875	8.25	8.15	1.02	108.25	57.1	49.05
2022/5/3	4982	53.0833	0	1.7	20.5708	12.6818	15.5455	1.0682	123.9545	68.9545	57.0909
2022/5/4	4651	51.125	0	2.5542	20.625	16.9474	11.8421	1.0947	142.4737	69.5789	60
2022/5/5	4269	62.7917	0	2.9458	20.8542	12.1364	7.5	0.9909	108.7727	31.3	23.2857
2022/5/6	4214	67.6667	0	2.5542	22.9208	13	6.125	1	99.25	27	9.1429
2022/5/7	3975	77.9167	0	2.6333	21.5375	12	8.0833	1.0083	61.25	26.1111	20.3333
2022/5/8	3947	62.9167	0	2.6417	20.525	5.6429	5.7143	1	88.7857	25.7857	16.7778
2022/5/9	3014	55.875	0	2.4625	18.7375	6.1176	6.5294	1.0059	96.2941	21.7143	13.0625
2022/5/10	1487	76.3333	0.0042	2.4625	18.8917	5.25	5.625	1.0188	86.375	27.4375	7.7778
2022/5/11	1449	78.0833	0	1.8583	19.9	5.3333	7	1.0333	81.6111	25.5	12.1111
2022/5/12	2069	91.25	0.4208	1.6292	18.3458	5.25	7.5	1.09	85.35	20.4	11.1111
2022/5/13	1681	87.5	0.45	1.9	16.0333	5.2353	6	1.1941	102	22.25	15.2353

2022/5/14	1369	59.7083	0	1.7042	17.8167	6	7.3333	1.2067	100.2667	34.7857	26.5333
2022/5/15	938	50.0833	0	1.5333	18.6917	6.3571	9.2143	1.1857	98.5714	27	21.5714
2022/5/16	823	48.625	0	2.225	20.55	8.1765	14.7059	1.2	94.8235	47	29.3529
2022/5/17	855	51.2083	0	2.3167	21.4958	9.4286	12.2381	1.3095	138.7143	55.0952	39.1905
2022/5/18	719	39.7917	0	2.7625	21.85	10.6	11.25	1.215	125.15	37.1667	22.8
2022/5/19	858	65.125	0.0083	2.7375	20.5375	7.3889	7.3889	1.2	126.8333	34	22.6667
2022/5/20	868	91.4167	0.3542	0.6833	19.1458	6.0556	13.2222	1.2	64.3889	30.9375	25.7778
2022/5/21	622	76.7083	0	1.4208	21.2125	6.2857	8.6667	1.1381	90.0476	20.5714	12.1579
2022/5/22	558	62.3333	0	3.3583	22.475	16.7647	8.4118	1.1765	106.7647	28.4118	16.1176
2022/5/23	480	66.7083	0	3.8458	21.275	8.45	5.8	1.175	108.4	19.65	8.9167
2022/5/24	387	91.7917	0.0583	2.5417	18.8	5.8571	5.6667	1.2	108	15.125	10.5789
2022/5/25	338	77.7917	0	1.4458	21.3083	6.5	10.8	0.845	93.75	18.7368	12.8947
2022/5/26	264	78.375	0.025	1.3792	20.7	4.5714	30.9286	0.6286	54.2857	31.7857	26
2022/5/27	170	73.2917	0	1.6542	22.8708	5.1739	25.8261	0.6913	119.6957	47.9565	37.5652
2022/5/28	122	79.9167	0.2292	3.3375	21.3542	6.5217	19.1304	0.7	107.7826	21.2273	17.3913
2022/5/29	67	95.0833	0.7	1.3958	21.6167	3.2273	22.5909	0.8045	63.7727	16.5263	13.4091
2022/5/30	31	76.375	0	1.7875	24.5125	4.0476	30.4286	1.0571	70.381	49.5238	42.0476
2022/5/31	15	47.3333	0	2.7792	22.9542	5.75	20.0833	0.5958	95.0833	16.3478	7.25
2022/6/1	13	83.5	0	2.3542	24.8042	7.0435	20.3478	0.2955	99.2174	34.7059	34.6957
2022/6/2	16	82.5833	0	2.6833	24.7333	6.5652	15.6957	0.1957	82.8696	17.8	45.087
2022/6/3	14	83.9167	0	3.4333	23.7125	6	11.1667	0.1458	82.375	15.9091	10
